# Advancing Tumor Treatment Through Artificial Intelligence and Mathematical Modeling: A Comprehensive Review

**DOI:** 10.1002/hsr2.72884

**Published:** 2026-07-27

**Authors:** Mohsin Kamran, Abdul Majeed, A S M Rafiul Haque, Johari Yap Abdullah

**Affiliations:** ^1^ Department of Mathematics Division of Science and Technology University of Education Lahore Pakistan; ^2^ Dental Anatomy Udayan Dental College Rajshahi Bangladesh; ^3^ Medical & Basic Dental Sciences, School of Dental Sciences, Health Campus Universiti Sains Malaysia Kota Bharu Kelantan Malaysia; ^4^ Oral & Maxillofacial Radiology, School of Dental Sciences, Health Campus Universiti Sains Malaysia Kota Bharu Kelantan Malaysia; ^5^ Dental Research Unit, Center for Transdisciplinary Research (CFTR), Saveetha Dental College, Saveetha Institute of Medical and Technical Sciences Saveetha University Chennai India

**Keywords:** artificial intelligence, deep learning, hybrid modeling, machine learning, mathematical modeling

## Abstract

**Background and Aims:**

Solid tumors emerge from uncontrolled cell division and interactions with neighboring cells that influence their growth and resistance to therapy. Anti‐cancer drugs are developed to disrupt these processes, but they often damage healthy cells, making treatment difficult to manage safely and effectively. As the global cancer burden continues to rise, there is an urgent need for more precise and personalized approaches to detection and therapy. The study's goal is to analyze recent breakthroughs in artificial intelligence (AI), hybrid frameworks, and mathematical modeling in tumor diagnosis and treatment, examine the challenges of data‐driven methodologies, and highlight emerging trends with future recommendations.

**Method:**

In recent years, computational methods like AI and mathematical modeling (MM) have emerged as powerful tools to address these challenges. AI techniques, including machine learning and deep learning, are increasingly used to improve early diagnosis, guide surgical planning, and predict treatment outcomes. Meanwhile, mathematical models offer valuable insights into tumor growth patterns and help optimize therapeutic strategies by simulating how tumors respond to different interventions.

**Results:**

AI and MM enhance cancer detection, prediction, and treatment through precise and optimized hybrid techniques. However, challenges such as inadequate data, poor interpretability, and generalization persist, necessitating better explainable and robust models for clinical use.

**Conclusion:**

This review explores the current applications of AI and MM in the field of neurosurgical oncology, emphasizing both their promising potential and the key limitations. It concludes by discussing future directions aimed at developing safer, more transparent, and individualized cancer care solutions that can ultimately improve patient outcomes.

**Abbreviations**
AI: artificial intelligence
ANN: artificial neural network
DL: deep learning
ER‐positive: estrogen receptor positive
FN: false negative
FP: false positive
FS: feature selection
KNN: K‐nearest neighbor
LQ: linear quadratic
ML: machine learning
MM: mathematical modeling
NN: neural network
RF: random forest
SVM: support vector machine
TN: true negative
TP: true positive

## 1 Introduction

**Figure 1:**
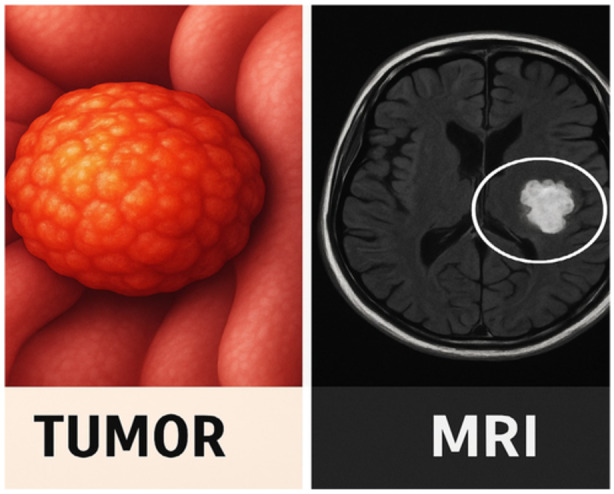
Visual of tumor cell and MRI image.

**Figure 2:**
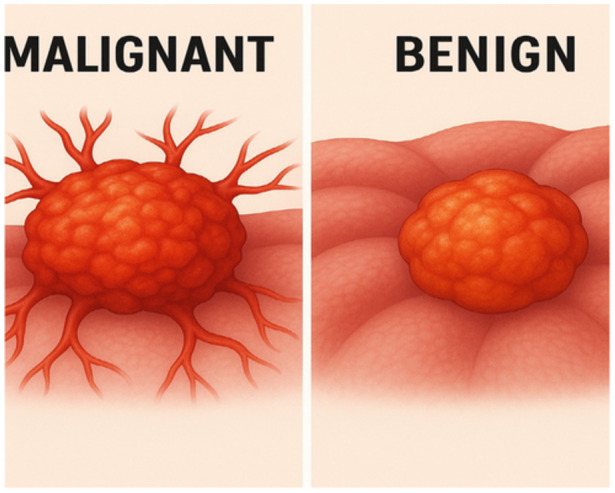
Classification of benign and malignant tumor.

Cancer is a complex and life‐threatening disorder characterized by abnormal cell growth that can arise in any organ. It was first recognized in the 1600s [1] as uncontrolled proliferation with the potential to invade distant sites through metastasis. Figure 1 shows tumor cells and their MRI depiction, while Figure 2 exhibits benign and malignant tumors.

Cancer cells are classed as benign or malignant. Benign cells do not spread to other organs, but malignant cells metastasis and do more damage. The disease's high mortality and recurrence rates make therapy time‐consuming and costly [2, 3]. Table S1 highlights the fundamental differences between cancerous and non‐cancerous tumors.

Cancer is a multifactorial genetic disorder resulting from cumulative genomic and epigenomic abnormalities that disrupt normal cellular regulation. Its progression is driven by interactions between malignant cells and the surrounding microenvironment, including immune and stromal components. Metabolic imbalance, cellular stress, and chromosomal instability further promote tumor development, while elevated glucose utilization supports rapid cell proliferation. Despite advances in diagnosis and treatment, many cancer types remain difficult to detect at an early stage. Therapeutic approaches include surgery, chemotherapy, radiotherapy, hormone therapy, and other targeted interventions, with screening strategies playing a key role in early diagnosis. In addition, molecular factors such as WNT16B have been linked to reduced responsiveness to anticancer treatments. Globally prevalent cancers include those of the breast, cervix, thyroid, lip, and oral cavity, whereas rare malignancies such as osteosarcoma, Ewing sarcoma, male breast cancer, gastrointestinal stromal tumors, chondrosarcoma, mesothelioma, chromophobe renal carcinoma, and ependymoma collectively account for approximately 20% of cancer cases [4, 5, 6]. Several microorganisms, including *Chlamydia trachomatis*, *Helicobacter pylori*, *Salmonella enterica*, and enterotoxigenic *Bacteroides fragilis*, have also been implicated in cancer development [7]. According to global cancer statistics, approximately 19.98 million new cancer cases were reported in 2022, and this number is projected to rise to 35.28 million by 2050, with an estimated 19.7 million cancer‐related deaths worldwide.

This study focuses on modern data‐driven methodologies, such as machine learning (ML), deep learning (DL), and hybrid mathematical modeling (MM) frameworks for advancing cancer research. It also addresses the limitations and challenges that artificial intelligence (AI) confronts in improving cancer and neurosurgery. Furthermore, this paper addresses future trends and directions. The manuscript is structured as follows: Section 1 provides an introduction, whereas Section 2 discusses hybrid modeling and treatment frameworks. Section 3 elaborates methodology utilized in this paper. Section 4 emphasizes the significance of AI in diagnosing and predicting tumors. Section 9 emphasizes discussion, followed by ethical concerns. Section 6 provides concluding remarks.

## 2 Hybrid Modeling

Hybrid discrete–continuous models combine physical laws with computational approaches to describe complex biological processes. Over the past decade, tumor modeling has increasingly integrated fluid dynamics, game theory, ML, and optimization techniques to improve predictions of tumor progression and treatment response [8]. The growing availability of preclinical and clinical data has further accelerated the adoption of data‐driven approaches. These approaches enhance model calibration, validation, and interpretation. Moreover, ML and computer‐aided methods are routinely applied to medical imaging modalities, including MRI, CT, PET, fluorescence imaging, and immunohistochemistry.

### 2.1 Modeling Chemotherapy

Chemotherapy employs antiproliferative agents to eradicate tumor cells or inhibit the growth and remains a cornerstone treatment for both localized and metastatic cancers. However, tumor heterogeneity, abnormal vasculature, elevated interstitial pressure, and the tumor microenvironment often limit drug delivery and therapeutic efficacy [9, 10]. In brain tumors, the blood–brain barrier further restricts chemotherapeutic transport, necessitating advanced modeling approaches to optimize treatment strategies [11]. Hybrid models have been developed to investigate tumor cell migration, drug resistance, and treatment outcomes, particularly in gliomas and non‐small cell lung cancer [12, 13, 14]. These studies demonstrate that microenvironmental heterogeneity can promote drug tolerance and influence chemotherapy effectiveness [13]. Comparative studies of nanoparticle‐based delivery systems have further improved treatment specificity and cytotoxicity [15]. Mathematical and optimal control models have also been applied to reduce systemic toxicity, evaluate tumor heterogeneity, optimize drug combinations, and improve dosing strategies [16, 17]. Additional investigations have explored synthetic anticancer compounds [18], PK/PD and Monte Carlo‐based scheduling approaches [19], translational PK‐tumor growth inhibition (TGI) frameworks for dose selection [20], PKPD models of tumor growth under therapy [21, 22], and image‐based predictive models for assessing responses to neoadjuvant chemotherapy [23].

### 2.2 Modeling Targeted Therapy

Targeted therapy selectively attacks cancer‐specific molecular abnormalities while minimizing damage to healthy tissues, thereby offering improved efficacy and reduced toxicity compared with conventional treatments [24]. Hybrid modeling approaches have been widely used to investigate targeted therapies, particularly those involving the MAPK and PI3K‐AKT signaling pathways in lung cancer and tyrosine kinase inhibitors in brain tumors [25, 26]. To overcome hypoxia‐induced treatment resistance, hypoxia‐activated prodrugs have been explored using spatially explicit PK/PD and multiscale modeling frameworks [27, 28, 29]. These studies demonstrated that optimizing drug activation, stability, and tumor oxygenation can significantly enhance therapeutic efficacy.

### 2.3 Modeling Nanotherapy

Nanotherapy employs nanoparticles to deliver therapeutic agents selectively to tumor sites, improving drug targeting while minimizing systemic toxicity. By exploiting tumor‐specific receptors and microenvironmental characteristics, nanoparticles can enhance drug accumulation and reduce adverse effects associated with conventional chemotherapy. Mathematical and hybrid modeling approaches have been used to optimize nanoparticle design, drug delivery, and treatment efficacy across multiple spatial and temporal scales [30, 31, 32]. To address complexities, optimization techniques, including continuous modeling, mesh adaptive direct search, and bi‐objective optimization, have been employed to identify nanoparticle properties that maximize drug accumulation and distribution [33, 34]. Furthermore, Bayesian hierarchical and statistical frameworks have been integrated with nanoparticle adhesion models to account for uncertainties in ligand‐receptor interactions and vascular characteristics, providing robust computational tools for the design of personalized nanotherapeutic strategies [35, 36].

### 2.4 Modeling Hormone Therapy

Hormone therapy is a systemic treatment designed to interfere with cells that depend on hormones for growth. Breast cancer (ER‐positive) and prostate cancer (AR‐positive) are two common examples of cancers that rely on hormones. Mathematical models have been established for these therapies; however, they are either continuous or based on game theory, as highlighted in [37, 38].

### 2.5 Modeling Immunotherapy

Immunotherapy enhances the body's immune response to recognize and eliminate tumor cells, either by stimulating immune activity in vivo or by improving immune cell function ex vivo before reinfusion into patients [39]. A major challenge is achieving sufficient immune cell infiltration throughout the tumor microenvironment. Computational and hybrid models have been developed to investigate immune–tumor interactions, immune cell distribution, and treatment efficacy [40, 41, 42]. Mathematical models have also been used to examine the impact of PD‐L1/PD‐1‐mediated immune suppression and tumor heterogeneity on T‐cell checkpoint therapies [42]. In addition, models of cytotoxic T‐lymphocyte dynamics have provided insights into tumor eradication mechanisms and immunization strategies [43]. Complementary in vitro, in vivo, and in silico studies further suggest that macrophage‐mediated drug delivery can improve therapeutic efficacy and promote sustained tumor reduction compared with conventional drug administration [44].

### 2.6 Modeling Radiotherapy

Radiotherapy is a localized cancer treatment that uses ionizing radiation to induce DNA damage and eliminate tumor cells, either as a standalone therapy or in combination with treatments such as chemotherapy [45]. Despite its clinical effectiveness, treatment resistance and tumor heterogeneity remain significant challenges. Mathematical, agent‐based, and hybrid modeling approaches have been widely employed to optimize radiation schedules, beam configurations, and patient‐specific treatment strategies [45], while cellular automata and continuous transport models have been applied to simulate tumor growth, oxygen dynamics, and radiation response [46]. Models incorporating heterogeneous cell populations have demonstrated that spatially varying radiation doses may outperform homogeneous treatment strategies in tumors with diverse radiosensitivity profiles [47]. Computational optimization techniques have also been used to improve beam intensity planning, minimize damage to surrounding organs, and account for patient‐specific uncertainties such as breathing motion [48]. Furthermore, robust optimization, ML, and adaptive Bayesian approaches have been integrated into radiotherapy planning to support PET‐guided treatments and predict individualized responses based on longitudinal patient data [49, 50, 51].

### 2.7 Modeling Combined Therapies

Tumor heterogeneity and the dynamic tumor microenvironment frequently promote drug resistance and disease recurrence, making combination therapies an attractive strategy for improving treatment outcomes. However, optimizing treatment sequences, dosages, and schedules remains challenging due to the large number of therapeutic variables involved. Mathematical and computational models have been developed to evaluate the combined effects of chemotherapy, immunotherapy, radiotherapy, and targeted treatments on tumor dynamics [52, 53, 54, 55, 56, 57]. Drug–disease and TGI models have also been used to predict treatment efficacy and overall survival based on longitudinal clinical data [53].

### 2.8 Modeling Adaptive Therapy

Cancer treatment strategies are traditionally selected based on tumor stage and grade at diagnosis; however, adaptive therapy dynamically adjusts treatment according to tumor response and progression to delay resistance and control specific tumor subpopulations [58]. Recent adaptive therapy frameworks increasingly integrate multiple mathematical approaches, with hybrid models demonstrating the potential to optimize multi‐drug treatment strategies in heterogeneous tumors [59]. The growing global burden of cancer underscores the need for more effective therapeutic interventions [60]. Although surgery, chemotherapy, radiotherapy, targeted therapy, and immunotherapy remain the primary treatment modalities [61, 62], their effectiveness is often limited in metastatic and advanced‐stage cancers [63]. Similarly, immunotherapy faces challenges related to immune checkpoint activation, inadequate antigen presentation, and tumor heterogeneity [64, 65]. These limitations have driven the development of computational approaches for cancer diagnosis, prognosis, and treatment optimization. Since the 1980s, computer‐aided methods have increasingly been adopted to improve diagnostic accuracy, reduce interpretation errors, and facilitate early cancer detection, which remains critical for improving patient outcomes [66, 67, 68, 69].

## 3 Methodology

This review was carried out to uncover relevant research breakthroughs in AI and hybrid modeling frameworks in neurosurgical oncology. The total number of articles searched, included, screened, and filtered was illustrated through the PRISMA flow diagram.

### 3.1 Literature Search Strategy

A thorough literature search was conducted using major scientific databases, including Google Scholar, Scopus, Web of Science, Science Hub, PubMed, Research gate, and arXiv. The search included papers published between 2013 and 2026, with seminal classical studies maintained for historical and theoretical context.

### 3.2 Inclusion Exclusion Criteria

The search included keywords such as “artificial intelligence,” “machine learning,” “deep learning,” “tumor,” “mathematical modeling,” “hybrid modeling,” “diagnosis,” “prediction,” “prognosis,” and “treatment optimization.” More than 400 articles were identified from 1995 to 2025. The study's inclusion criteria were peer‐reviewed journal publishing, English language, and a focus on search keyword‐based content research. Among these articles, emphasizes was on the topics related to keywords with comprehensive methodological descriptions and robust scientific evidence. Conversely, studies lacking peer‐review, insufficient methodological and clinical material and information, and/or duplicate studies were excluded, resulting in approximately 300 screened articles being included.

### 3.3 Study Selection and Data Collection

The obtained records were reviewed for titles and abstracts, and possibly relevant publications were assessed using full‐text evaluations based on preset eligibility criteria. Before final selection, duplicate studies were excluded, and for each eligible record, key information, including methodology, tumor site, clinical data, outcomes, and limitations were extracted for analysis.

### 3.4 Data Analysis and Synthesis

Finally, selected studies were systematically categorized into several core themes:

- Development of mathematical models for tumor progression and AI‐based cancer diagnosis.
- Deep and ML applications
- Hybrid mathematical frameworks
- Optimization strategies of cancer prognosis and treatments.
- Challenges and future directions.

Tabular and graphical summaries were presented to understand the accuracy measures of applied algorithms to treat various cancers.

### 3.5 Reproducibility

**Figure 3:**
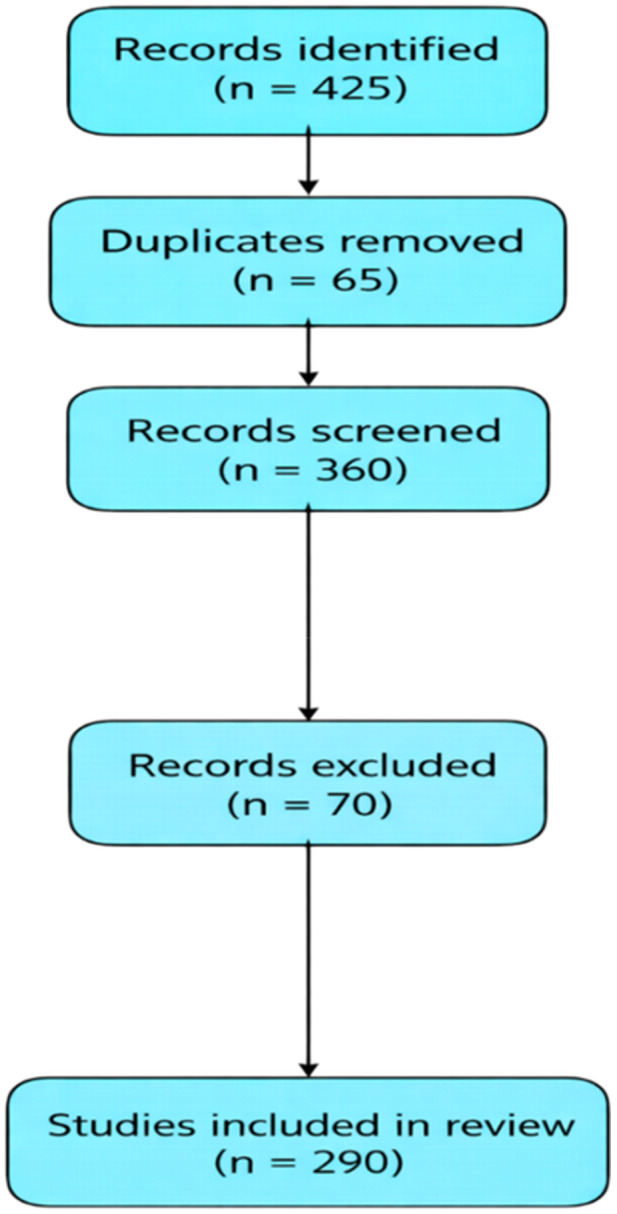
PRISMA flow diagram.

To enhance reproducibility, the methodology states the search databases, inclusion criteria, screening, and eligibility process illustrated through the PRISMA flow diagram as displayed in Figure 3.

## 4 AI, ML, DL, and MM

### 4.1 AI

**Figure 4:**
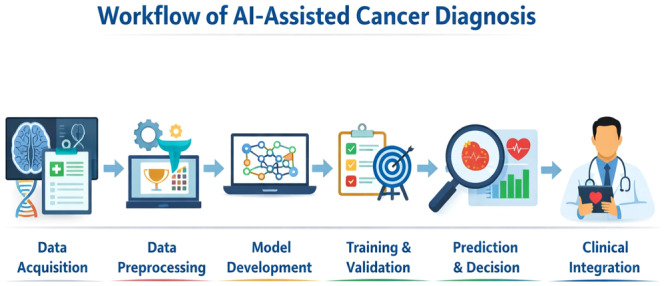
Workflow of AI‐assisted cancer diagnosis.

AI encompasses computational techniques that enable machines to perform tasks requiring human‐like reasoning, learning, and decision‐making. In oncology, AI has emerged as a powerful tool for enhancing tumor diagnosis, prognosis, and treatment planning, particularly in the management of brain tumors and neurosurgical applications [70]. By analyzing large‐scale clinical and imaging datasets, AI algorithms can identify subtle patterns that may not be readily detectable by clinicians, thereby improving diagnostic accuracy, early detection, and patient outcomes. Automated image analysis further reduces clinical workload by efficiently identifying regions of interest and supporting decision‐making processes. Since biomedical data are often heterogeneous and unstructured, effective data preprocessing is essential for converting raw data into formats suitable for ML applications. Common preprocessing steps include data reduction, projection, and handling of missing values [71, 72]. The general workflow of AI‐assisted cancer diagnosis is illustrated in Figure 4.

### 4.2 AI‐Based Cancer Diagnosis

AI‐based systems are widely applied to medical imaging modalities such as MRI, CT, and PET for tumor detection, classification, and segmentation [73, 74, 75, 76, 77, 78]. DL models, particularly convolutional neural networks (CNNs), have demonstrated high accuracy in identifying complex imaging patterns and improving the early detection of lung, breast, brain, and other cancers [74, 76]. Traditional ML techniques, including support vector machines (SVMs), decision trees (DTs), and artificial neural networks (ANNs), continue to support feature extraction and classification tasks, often complementing DL approaches. By automating image analysis, AI reduces human error, inter‐observer variability, and diagnostic workload while enhancing clinical decision‐making and patient outcomes [77]. Recent studies have further highlighted the integration of AI with mechanistic models for cancer detection, treatment planning, and drug discovery [79]. AI‐assisted frameworks have also shown promise in precision oncology, supporting cancer screening, risk stratification, diagnosis, and treatment‐response prediction, with several tools already undergoing clinical evaluation and regulatory approval [80]. Moreover, comprehensive reviews have emphasized the growing role of AI‐driven strategies in early cancer detection, diagnosis, and treatment across multiple malignancies [81]. Despite these advances, challenges related to data availability, model interpretability, overfitting, data imbalance, and generalizability continue to limit widespread clinical implementation [82, 83].

### 4.3 AI in Treatment Prediction and Prognosis

Beyond diagnosis, AI plays a pivotal role in cancer prognosis and treatment prediction by providing data‐driven insights into disease progression and therapeutic response. ML models have been widely employed to predict patient survival, tumor recurrence, and responses to chemotherapy, radiotherapy, and immunotherapy by integrating clinical, genomic, and imaging data [84, 85, 86, 87, 88]. These approaches facilitate personalized medicine by supporting treatment selection tailored to individual patient characteristics [87, 88]. Nevertheless, challenges related to data quality, dataset bias, model interpretability, and generalizability remain significant barriers to the widespread clinical adoption of AI‐based predictive systems [89, 90].

### 4.4 MM of Tumor Growth

MM provides a mechanistic framework for understanding tumor growth, progression, and treatment response by representing complex biological processes through mathematical formulations [91, 92, 93]. Classical growth models, including exponential, logistic, and Gompertz models, describe different patterns of tumor development, with the latter two incorporating environmental constraints such as nutrient availability and immune regulation [93]. More advanced models integrate spatial heterogeneity, angiogenesis, immune interactions, and drug dynamics to better capture tumor behavior and therapeutic outcomes [94, 95]. Despite their utility, these models are often limited by challenges in parameter estimation, biological variability, and simplifying assumptions that may not fully represent tumor complexity and patient‐specific heterogeneity.

### 4.5 Hybrid AI‐Mathematical Models

The integration of AI with MM has emerged as a promising approach in cancer research, combining data‐driven learning with mechanistic understanding [95, 96, 97, 98, 99]. Hybrid frameworks employ ML to improve parameter estimation, model calibration, and predictive accuracy, while mathematical models provide biological interpretability. By integrating clinical and experimental data with mechanistic formulations, these approaches enable more accurate predictions of tumor growth, disease progression, and treatment response. Hybrid models are particularly valuable for personalized medicine, supporting adaptive therapy planning, optimization of drug combinations, and treatment scheduling.

### 4.6 ML

**Figure 5:**
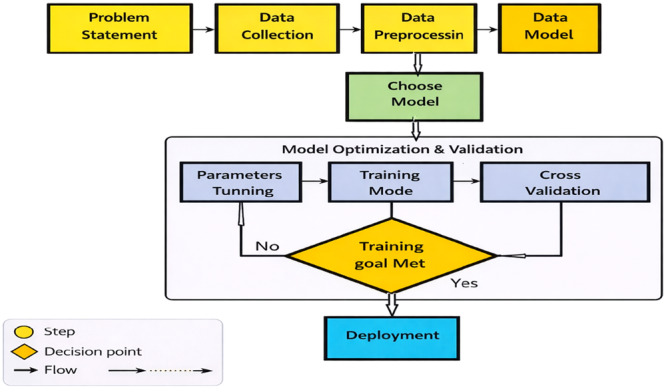
Flowchart illustrating the machine learning workflow in healthcare, starting from defining the problem statement and progressing through data collection, preprocessing, model optimization, validation, and ending in deployment.

ML, a core branch of AI, enables computational systems to learn patterns from data and has become an essential tool in biomedical research and oncology [100, 101]. ML techniques support disease risk assessment, prognosis, classification, and prediction by analyzing large‐scale clinical, genomic, and imaging datasets [102, 103, 104]. The ML workflow generally involves data collection, preprocessing, model training, validation, and deployment, as illustrated in Figure 5.

**Figure 6:**
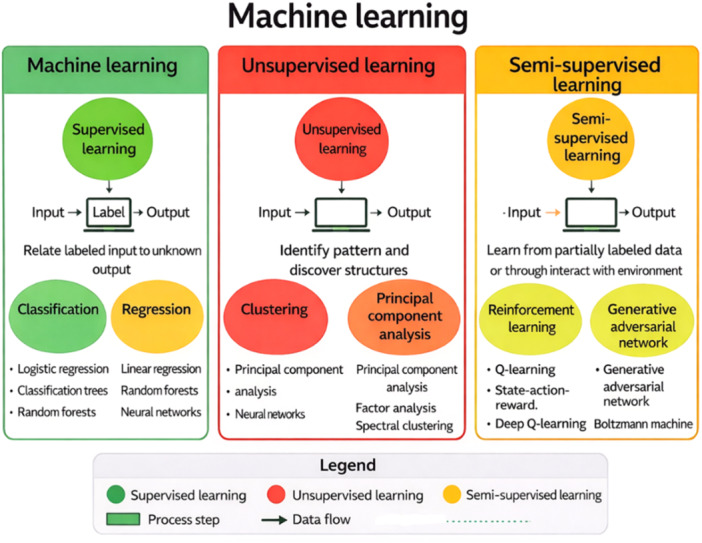
Overview of machine learning paradigms in healthcare, categorizing supervised, unsupervised, and semi‐supervised learning, including examples and process steps.

Supervised learning uses labeled data to predict outcomes such as patient survival, whereas unsupervised learning identifies hidden structures and clusters within biomedical data, including radiomics applications [105, 106, 107, 108, 109, 110, 111]. Reinforcement learning, which optimizes decisions through reward‐based mechanisms, has also shown promise in drug discovery, molecular design, and personalized treatment strategies [107, 112, 113, 114]. An overview of major ML paradigms and their applications is presented in Figure 6.

Common ML algorithms include neural networks (NN), k‐nearest neighbors (KNN), naïve Bayes, DT, SVM, and random forests (RF), each offering distinct advantages for classification and predictive modeling [101, 115, 116]. Since its inception in 1958, ML has evolved into a key component of modern healthcare, enhancing clinical decision‐making and enabling early cancer detection through autonomous diagnostic systems [100, 101, 102, 117, 118, 119, 120, 121].

Moreover, ML facilitates personalized medicine by predicting treatment outcomes and identifying clinically relevant patterns from complex biomedical data [67, 122, 123, 124]. Recent studies have integrated ML with optimization techniques to improve predictive performance. For example, XGBoost, ANNs, and graph neural networks have been applied to carcinoma prediction [125], while genetic algorithm (GA)‐assisted ML models have enhanced feature selection and breast cancer diagnosis [126]. Similarly, DL combined with GA‐based optimization has achieved high accuracy in lung cancer detection [127]. Since model performance depends heavily on data quality, approximately 80% of ML workflows are devoted to data collection and preprocessing [128, 129], requiring comprehensive clinical datasets that include imaging, laboratory findings, treatment histories, and follow‐up outcomes [118, 120, 130, 131, 132, 133]. Numerous investigations have demonstrated the effectiveness of ANN, SVM, DT, and hybrid ML models in cancer prediction [134, 135, 136, 137], while ANNs have shown particular strength in medical image analysis due to their multilayered feature‐learning capabilities [71, 138]. Recent work has also highlighted the growing role of mechanistic learning in bridging data‐driven and mathematical approaches for oncology applications [139]. Comparative summaries of AI, ML, and DL methods are presented in Tables S2 and S3.

### 4.7 ML Algorithms in Specific Cancers

#### 4.7.1 Glioma

ML has become increasingly important in glioma research, particularly for tumor grading and classification using medical imaging. Studies utilizing MRI sequences such as T1W, T2W, and FLAIR have demonstrated the effectiveness of ML techniques, with RF and least absolute shrinkage and selection operator models achieving comparable performance in distinguishing low‐ and high‐grade gliomas [140]. Kibriya [141] reported that their fused feature approach, trained on 15,320 MR images, outperformed individual feature vectors and previous methods, achieving an accuracy of 99.7%.

#### 4.7.2 Oral Cancer

Early detection and accurate prognosis are critical for improving oral cancer management and patient survival. ML has emerged as a valuable tool for enhancing diagnostic accuracy and predicting cancer susceptibility, recurrence, and survival outcomes [142, 143, 144, 145, 146]. Advanced imaging approaches have demonstrated significant potential; for instance, a comparative study showed that 3D CNNs outperformed 2D CNNs in distinguishing benign from malignant oral lesions by utilizing dynamic enhancement rate features [147]. Furthermore, ANN models integrating risk factors, general health information, and clinicopathological characteristics have achieved promising predictive performance, with reported accuracies approaching 79% for oral cancer risk assessment [148].

#### 4.7.3 Skin Cancer

Early melanoma diagnosis remains challenging, making computer‐aided approaches increasingly valuable in clinical practice. Advances in image processing and machine vision have expanded the use of supplementary imaging for skin cancer diagnosis and surgical planning [149, 150]. Publicly available datasets, such as the PH2 dataset and the ISIC archive, have accelerated the development and evaluation of ML models for skin lesion classification and segmentation [151, 152, 153]. For example, SegNet achieved 94% accuracy on dermoscopic images from the PH2 dataset [154], while multi‐resolution CNN ensembles further improved melanoma classification by exploiting features at multiple image scales [155]. Recent AI developments, including attention mechanisms, image enhancement techniques, and hybrid CNN–Vision Transformer architectures, have enhanced classification performance by capturing both local and global image features [78, 156, 157, 158, 159]. In addition, explainable AI and multimodal integration have been increasingly adopted to improve the interpretability, reliability, and clinical applicability of skin cancer diagnosis systems [160].

#### 4.7.4 Pancreatic Cancer

Distinguishing pancreatic cancer from benign disorders can be difficult. Studies show that ML models such as k‐NN, ANN, SVM, LR, and RF can extract unique imaging features useful for pancreatic cancer diagnosis [161, 162].

#### 4.7.5 Colon Cancer

Colonoscopy remains the main diagnostic approach for colon cancer, though it is invasive and resource‐limited. Diagnosis is further complicated by interactions between patients, physicians, and healthcare technologies [163]. Several datasets are also employed for colon cancer detection [164].

#### 4.7.6 Lung Cancer

Lung cancer is the leading cause of cancer deaths, affecting 29.2% of men aged 45%−64% and 22.8% over 65, while in women the rates are 17.9% and 13.7%, respectively [165]. Diagnosis involves methods such as CT scans, bronchoscopy, sputum cytology, thoracoscopy, and lymph node biopsy [166]. Early lung cancer detection often combines patient clinical data (age, gender, family history, smoking, etc.) with CT nodule features (size, type, location, count, borders, emphysema) to train ML models like LR or LDA [167, 168]. Another study tested six classifiers, including SVM, RF, k‐NN, and AdaBoost, using metabolomic biomarkers for improved tumor prediction. Li et al. [169] compiled studies from various researchers that applied different ML models and classifiers for early cancer detection and diagnosis.

### 4.8 Performance Metrics

Performance measurements are an integral aspect of any ML pipeline. The well‐known accuracy measures are presented here [70].

#### 4.8.1 Confusion Matrix

A confusion matrix is a table that displays the various predictions and categorization results. It aids in visualizing the outcome as well as identifying the proper forecasts for various sorts of errors. The four main parts of it are false positive (FP), false negative (FN), true positive (TP), and true negative (TN) [70].

#### 4.8.2 Classification Accuracy

The most common performance parameter for classification models is accuracy. This metric measures the frequency with which values are correctly classified. Mathematically, it is calculated as [70]:

$$\text{Accuracy}\;=\frac{\text{TP}+\text{TN}}{\text{TP}\;+\;\text{FP}+\text{FN}\;+\;\text{FP}}$$

where TPs refer to the number of times real positive values match projected positive values. TNs show how often the actual negative value matches the projected negative value, and FP occur when a model incorrectly forecasts negative values as positives. FN refers to the model's incorrect prediction of positive values as negative.

### 4.9 Classification Report

1. Precision: Precision determines how accurate the model is at correctly classifying positive values. “When the model predicts a positive value, how often is it correct” it asks. Mathematically,
$$\text{Precision}\;=\;\frac{\mathrm{TP}}{\mathrm{TP}+\mathrm{FP}}$$
2. Recall or sensitivity: Recall refers to the model's ability to anticipate positive results. “How often does the model predict the correct positive value.” Mathematically,
$$\text{Precision}\;=\;\frac{\mathrm{TP}}{\mathrm{TP}+\mathrm{FN}}$$
3. Specificity: Specificity refers to the number of negative results returned by our ML model.
  Mathematically,
$$\text{Precision}\;=\;\frac{\mathrm{TN}}{\mathrm{TN}+\mathrm{FP}}$$
4. Support: Support refers to the number of true responses in each target value class.
5. F1‐Score/F‐measure: The F1‐score is a weighted average of precision and recall, calculated as the harmonic mean of both.

Mathematically,

$$F1-\text{Score}=\frac{2\times \text{Precision}\times \;\text{Recall}}{\text{Precision}+\text{Recall}}$$

#### 4.9.1 Cohen's Kappa: (κ)

Cohen's Kappa is a statistical metric used to assess the agreement between two raters (or a model vs. ground truth) for categorical data, while accounting for chance agreement [170].

Mathematically,

$$\kappa =\frac{P_{o}-P_{e}}{1-P_{e}}$$

here,

$$P_{0}=\text{Observed value}=\frac{\mathrm{TP}+\mathrm{TN}}{N}$$

$$P_{e}=\text{Estimated value}=\frac{\left(\mathrm{TP}+\mathrm{FP}\right)\;\left(\mathrm{TP}+\mathrm{FN}\right)+(\mathrm{FN}+\mathrm{TN})\;(\mathrm{FP}+\mathrm{TN})}{N^{2}}$$

6. Balanced accuracy: Balanced accuracy is a performance metric used especially for imbalanced datasets,

which computes the average of sensitivity (recall) and specificity [171]. Mathematically,

$$\text{Balanced Accuracy}\;=\frac{\text{Sensitivity}+\text{Specivicity}\;}{2}$$

where, $\text{Sensitivity}\;=\;\frac{\mathrm{TP}}{\mathrm{TP}+\mathrm{FN}}$ and $\text{Specificity}\;=\;\frac{\mathrm{TN}}{\mathrm{TN}+\mathrm{FP}}$

### 4.10 DL

**Figure 7:**
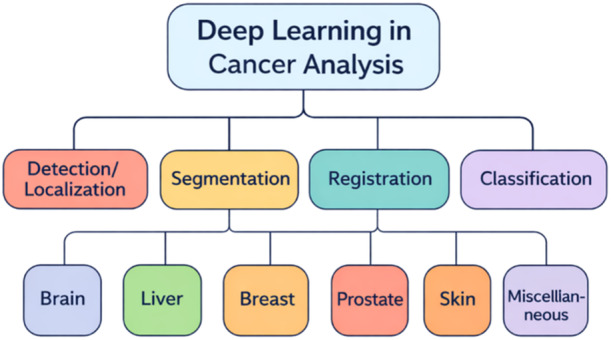
Applications of deep learning in cancer analysis, including detection/localization, segmentation, registration, and classification of various cancer types.

DL, a specialized branch of ML, has become a powerful tool for image recognition, natural language processing, disease diagnosis, drug discovery, precision medicine, and clinical decision support [101, 172, 173]. Unlike conventional AI methods that rely on sequential processes such as data preprocessing, feature extraction, feature selection, and model training [69], DL automatically learns hierarchical features directly from raw data, improving both efficiency and predictive performance [174]. Numerous studies have demonstrated the effectiveness of DL in oncology. Deep belief networks have been applied to three‐dimensional brain imaging with reduced computational complexity [175], while interpretable and targeted DL approaches have improved breast cancer diagnosis [176, 177]. GoogleNet‐based CNN models have enhanced lung cancer detection from CT images [178, 179], and CNN frameworks have achieved reliable performance in skin cancer classification [180]. Deep NNs have also achieved approximately 99.73% accuracy in breast cancer classification, whereas CNN‐based thermal imaging systems have improved breast disease diagnosis [181, 182]. Recent investigations have further explored multimodal imaging, radio genomics, and brain tumor segmentation using advanced DL frameworks [183, 184]. Figure 7 summarizes the application of DL in medical image analysis for cancer detection.

**Figure 8:**
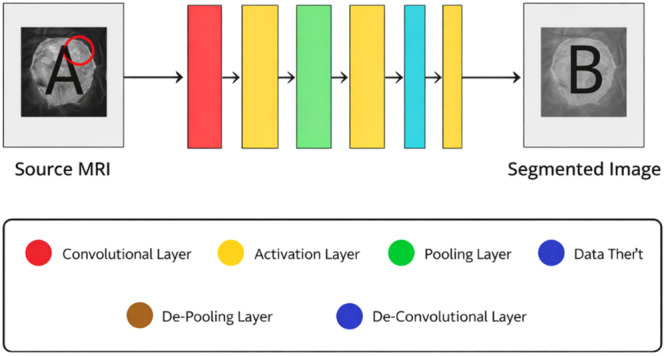
Typical convolutional neural network (CNN)‐based medical image segmentation framework illustrating the processes from source MRI to segmented image with legends for various neural network layers.

Figure 8 illustrates a typical CNN‐based segmentation framework. DL has also shown promising results in thyroid and brain tumor analysis. ANN‐based methods have been applied to thyroid nodule classification [185], while multimodal DL frameworks combined with optimization techniques have enhanced brain tumor classification using MRI data [186]. Automated CNN design through neural architecture search and hybrid optimization strategies involving GAs [187, 188] further improve model efficiency. In addition, DL‐assisted analysis of whole‐slide cytopathology images supports thyroid cancer diagnosis [189], whereas CNN and RF models have demonstrated reliable brain tumor segmentation with Dice coefficients of 0.876 and 0.862, respectively [190, 191]. Hybrid classifiers have also improved mammography‐based breast cancer detection [192].

Modern architectures such as GoogLeNet, ResNet, and Xception effectively capture multiscale image features while reducing parameter complexity [193, 194]. Moreover, long short‐term memory networks enhance learning from sequential data through specialized memory mechanisms [195], and transfer learning enables pre‐trained CNNs to be adapted to new tasks with limited labeled datasets, improving generalization and reducing training costs in medical imaging applications [196, 197].

#### 4.10.1 Publicly Available Datasets for Cancer Research

A quick overview of publicly available cancer datasets is presented in Table S4 along with [7].

### 4.11 MM

MM provides a systematic framework for representing biological processes and predicting disease dynamics [198]. Its application in medicine predates ML, beginning with Bernoulli's pioneering work on smallpox vaccination [199], and has expanded considerably since the 1990s, particularly in oncology [200, 201]. MM has been widely used to describe tumor growth and treatment response, with models such as the exponential, logistic, and Gompertz formulations capturing different growth behaviors [202]. The mathematical representation of cancer incidence in multistage models can be expressed as:

$$I=\frac{N_{q_{1}.q_{2}.q_{3}.,\ldots ..,q_{r}}}{\left(r-1\right)!}t^{r-1}$$

$$I=N_{q_{1}}\{1-e^{q_{k}^{2}}\left(e^{\mathrm{kt}}-1\right)\}$$

where *k* is a constant, *N* denotes the number of cells at risk, *r* represents the number of mutational changes, *I* is the cancer incidence, and *q* corresponds to the probability of cellular change at a given age. Foundational theories, including the Armitage–Doll multistage model and the linear‐quadratic (LQ) model, have advanced the understanding of carcinogenesis and radiotherapy response [203, 204, 205, 206], although their simplifying assumptions limit their ability to fully represent tumor complexity [207]. Figure S1 and Table S5 summarize mathematical advancements in oncology and modeling frameworks.

Mathematical models of tumor angiogenesis have incorporated angiogenic factors, capillary dynamics, and vascular growth to evaluate antiangiogenic therapies [208, 209, 210, 211, 212], while reaction‐diffusion frameworks have been widely used to study glioma growth and metastasis [213, 214, 215, 216]. In addition, MM supports the optimization of chemotherapy, radiotherapy, and surgical interventions through biologically effective dose and LQ‐based formulations [217, 218, 219, 220, 221, 222, 223]. Recent studies have further explored tumor growth kinetics, intratumor heterogeneity, personalized medicine, clinical trial optimization, and cancer immunology [224], with GA‐based optimization playing an increasingly important role in cancer diagnosis and treatment prediction [225, 226, 227, 228]. Optimal control strategies to minimize adverse effects and maximize therapeutic outcomes and stochasticity have been discussed in [229, 230].

**Figure 9:**
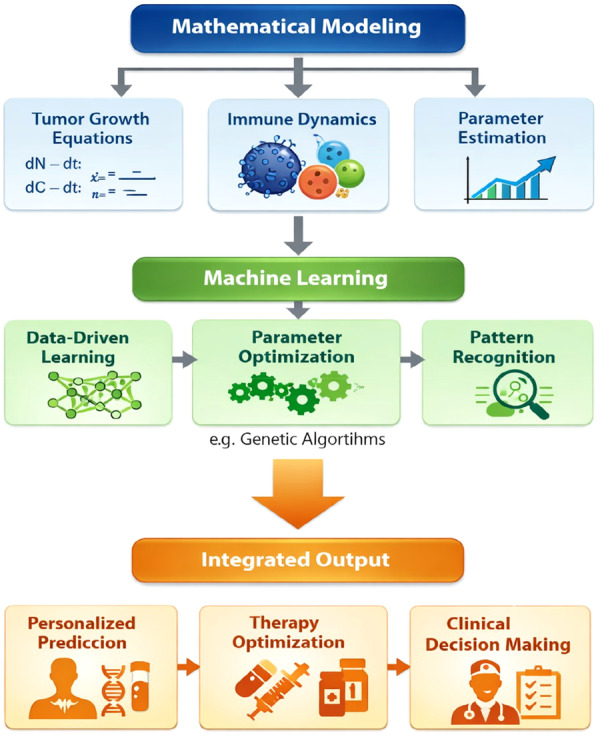
Pictorial view of integration of mathematical models with machine learning.

**Figure 10:**
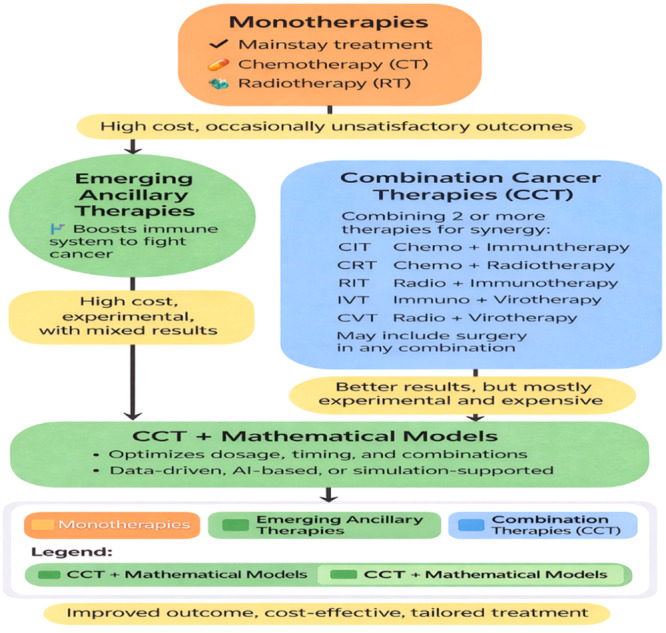
Flowchart illustrating various cancer therapy approaches: monotherapies, emerging ancillary therapies, combination cancer therapies (CCT), and the integration of CCT with mathematical models to optimize treatment.

MM has further advanced the understanding of drug resistance, therapeutic response, and treatment scheduling. Studies have examined solid tumor dynamics [231], hormesis and antibody dosing [232], drug delivery barriers in vascular tumors [233, 234], radiation treatment optimization [235], DNA methylation dynamics [236], and GA‐based chemotherapy optimization [237]. Comprehensive reviews of tumor progression models [238, 239], ODE‐based xenograft models [240], and pharmacokinetic‐pharmacodynamic frameworks [212, 241, 242, 243] further demonstrate the broad applicability of MM in oncology. Recent developments increasingly integrate ML with MM to improve parameter estimation, predictive accuracy, and treatment optimization [244, 245, 246, 247, 248]. Hybrid frameworks combining ML and MM have shown enhanced therapeutic performance and cost‐effectiveness, as illustrated in Figures 9 and 10. In brain tumor research, comparative analyses of ML classifiers, including SVM, KNN, CNN, RF, VGG19, AlexNet, GoogleNet, CapsNet, and GBM, have demonstrated the superior performance of CNN‐based approaches [66]. Moreover, feature selection techniques remain fundamental for cancer prediction, prognosis, and tumor growth modeling by identifying clinically relevant predictive factors [249].

## 5 Discussion and Future Directions

This review highlights recent research efforts that use AI‐based learning to predict malignancy. Although cancer research is progressing day by day by using data‐driven methodologies such as “AI, ML, DL, MM” and hybrid models. As the use of AI in neurosurgery grows, evaluating new programs will become increasingly important. Evaluations should consider patient and clinician acceptance, clinical efficacy, and ethical considerations.

### 5.1 Challenge and Suggestions

Despite significant progress, the application of AI and MM in oncology faces several challenges. Many models depend on large, diverse, and well‐annotated datasets for robust training and validation [82, 83]. Model interpretability, particularly in DL systems, remains a major concern, as clinicians require transparent and explainable predictions for informed decision‐making [89, 90]. Moreover, reproducibility, generalizability across different populations, and real‐world validation are essential for reliable clinical implementation [250]. The development of effective ML tools largely depends on the availability of high‐quality training data. Missing values, often caused by incomplete questionnaires or heterogeneous data collection, remain a major challenge and can substantially reduce model performance [251, 252]. In addition, biases arising from retrospective and observational datasets may lead to unreliable predictions and limited applicability in diverse clinical settings [253]. Therefore, the successful implementation of ML‐based prediction systems requires close collaboration between healthcare professionals and computational scientists to ensure appropriate data analysis, model development, and clinical interpretation [254].

**Figure 11:**
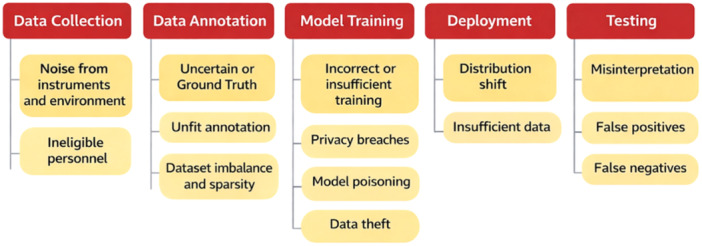
Major challenges and ethical risks associated with machine learning applications in healthcare, including issues related to data collection, annotation, model training, deployment, and testing.

MM complements data‐driven approaches by simplifying biological processes into mathematical formulations and evaluating them through numerical simulations [255]. However, parameter estimation, biological variability, and limited clinical data often restrict the ability of these models to accurately represent complex therapeutic scenarios, such as combined chemotherapy and antiangiogenic treatments [256]. Similarly, ML performance is highly dependent on both the quantity and quality of data, with factors such as model complexity, label availability, and data heterogeneity influencing predictive accuracy [257, 258, 259, 260, 261]. Medical applications are particularly challenging because of the diverse nature of clinical data, including genomics, imaging, laboratory tests, and physiological measurements [260]. Figure 11 summarizes the major challenges associated with ML applications in oncology [262].

**Figure 12:**
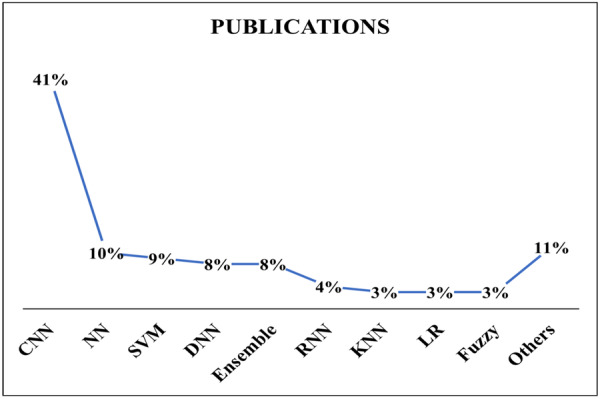
Distribution of publications among various ML techniques.

**Figure 13:**
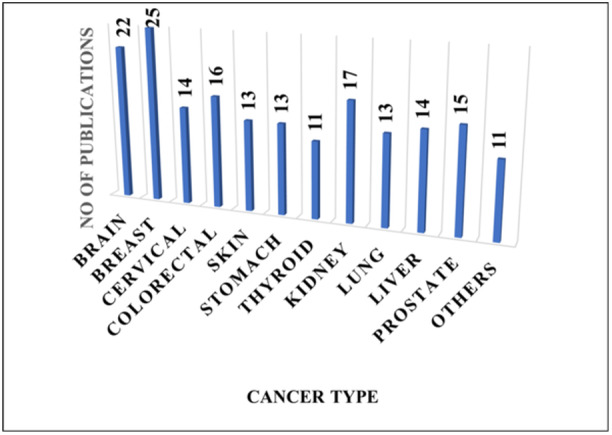
No of publications for various cancer sites.

Ensemble learning techniques, including RF, GBM, and SVM, are also widely used. Figures 12 and 13 illustrate the distribution of AI‐based prediction methods and their applications across different cancer types.

MRI remains the most frequently used imaging modality, followed by CT, dermoscopic, mammographic, endoscopic, and pathological images. CNNs have been extensively applied to brain, colon, skin, thyroid, and lung cancers, whereas hybrid approaches are common in breast cancer prediction [263, 264]. Nevertheless, DL models require large annotated datasets to avoid overfitting and achieve robust performance [264]. Public understanding and acceptance are also important for the successful adoption of AI in healthcare. Studies indicate that awareness of AI remains limited, emphasizing the need for transparent communication and gradual integration of AI‐assisted systems into clinical practice [265]. To improve public trust, it has been recommended that AI technologies support rather than replace clinical decision‐making, that their benefits and limitations be communicated openly, and that validation data be publicly available [265]. Overall, AI‐based models, including ANN, SVM, and hybrid frameworks, offer strong predictive performance and the ability to analyze high‐dimensional data for early tumor detection. However, they remain dependent on large, high‐quality datasets and are affected by overfitting, data imbalance, limited interpretability, and sensitivity to noise. Likewise, mathematical tumor models provide valuable mechanistic insights but often rely on simplifying assumptions and uncertain parameter estimation, limiting their ability to capture biological complexity. Reproducibility remains a significant concern because many studies are based on small or institution‐specific datasets without external validation, while variations in data acquisition, preprocessing, and model training further restrict the generalizability of AI and mathematical models across diverse patient populations.

### 5.2 Ethical Concerns

Beyond the methodological aspects of ML and MM, ethical and legal considerations are essential for their responsible application in healthcare. AI‐based systems may suffer from limited reproducibility and fairness when trained on datasets that underrepresent specific patient populations. Key ethical concerns include data security and confidentiality, algorithmic bias, trust relationships, accountability, physician–patient autonomy, and the potential impact of automated decision‐making on clinical practice [266]. The application of ML for predicting mortality in pediatric intensive care units further illustrates these challenges, as biases and inaccuracies in training data may influence treatment decisions. Ethical issues also arise when clinicians and healthcare systems rely heavily on algorithm‐generated risk predictions. Ideally, risk calculators should incorporate patient‐specific information to support informed consent and shared decision‐making. Otherwise, excessive reliance on such tools may reduce patient‐centered care, limit therapeutic choices, and encourage paternalistic decision‐making in high‐risk situations. Moreover, insurance providers and healthcare programs may use risk calculators to determine eligibility for treatment or evaluate physician performance, potentially bypassing the patient–physician relationship and penalizing clinicians whose decisions fall outside predefined risk thresholds [267].

Although ML has considerable potential to improve clinical outcomes, its integration into healthcare requires addressing four major ethical principles: informed consent, safety and transparency, algorithmic fairness and bias mitigation, and data privacy [268, 269]. The legal and policy implications of ML‐based healthcare systems continue to generate debate, emphasizing the need for regulatory frameworks that enable policymakers to address emerging ethical challenges associated with the growing adoption of AI in medicine [269, 270]. In addition, ML algorithms imitate aspects of human decision‐making but possess limited reasoning capabilities, creating further ethical concerns. The Medical Internet of Things also introduces security and interference challenges associated with data transmission and connectivity [271, 272].

Regulatory policies play a critical role in the sustainable development of AI technologies, requiring algorithm developers across scientific disciplines to address evolving legal standards [273, 274]. Data privacy remains a major concern because ML models rely on large volumes of patient information that may be stored across multiple locations, increasing the risk of unauthorized access and cyberattacks [275]. Protecting patient confidentiality, therefore requires transparent data collection and usage policies, informed consent procedures, and safeguards against unlawful exploitation of personal information. To enhance security, recent studies have proposed non‐invasive and secure DL‐based cancer diagnosis frameworks that encrypt medical data during transmission. The effectiveness of these approaches has been evaluated using security measures such as correlation, entropy, contrast, structural content, and energy, demonstrating their potential to reduce the risk of data theft.

### 5.3 Practical Implications

This study found that AI, ML, DL, and MM have the potential to greatly enhance cancer diagnosis, prognosis, and treatment planning. AI‐powered techniques can assist clinicians in detecting tumors at an earlier stage, improving diagnostic accuracy, reducing interpretation time, and enabling personalized therapeutic options. Mathematical and hybrid models bring value by simulating tumor development dynamics, forecasting treatment responses, and optimizing treatment regimens. Integrating computational technologies into clinical operations may enhance precision medicine, reduce healthcare costs, and optimize patient outcomes.

### 5.4 Limitations

Despite the comprehensive breadth of this assessment, limitations must be First, only research published in English was examined, potentially leading to linguistic bias. Second, the literature assessment indicated considerable disparities in datasets, cancer types, computational methodologies, validation strategies, and performance measures, complicating direct comparisons. Third, many AI‐based studies used retrospective datasets with no external validation, restricting their applicability to a wide range of clinical populations. Finally, while many computational models perform well in research settings, their use in everyday clinical practice is limited due to challenges with interpretability, regulatory approval, data protection, and clinical validation.

### 5.5 Future Directions

Future research should focus on creating explainable, trustworthy systems that provide clinicians with transparent decision‐making tools. To improve prediction performance and personalized treatment planning, multimodal data sources such as imaging, genomic, pathology, and clinical information should be better linked. Federated learning frameworks could help address data privacy concerns while also enabling collaborative model training across institutions. Furthermore, digital twin technologies and patient‐specific models provide opportunities for simulating sickness development and improving therapy approaches in real time. Large‐scale prospective clinical trials, as well as external validation across diverse populations, are necessary to assess the dependability, robustness, and clinical utility of AI‐driven and hybrid computational methodologies in oncology. To translate these advances into real healthcare solutions, physicians, mathematicians, computer scientists, and biomedical researchers must continue to collaborate.

## 6 Conclusion

This study reviews advanced cancer detection methods, emphasizing the role of ML, DL, and MM in early diagnosis through medical imaging. These approaches help identify disease signals, predict cancer risk, recurrence, and survival, and design patient‐ specific treatment plans based on genetic and clinical data. Unlike traditional lab testing, which is costly and time‐intensive, computational techniques streamline analysis, making cancer management more efficient and precise.

Our findings show that most prior work relies on ML and DL, particularly CNNs, where pre‐processed and segmented images outperform other metrics. However, early detection of head and neck cancers still requires further study. Major challenges include high‐dimensional and imbalanced datasets, missing data, and limited samples. Future research should focus on strengthening DL models for diagnosis, prognosis, and treatment prediction. In brain tumor surgery, hybrid modeling can support more effective therapy planning and guide personalized clinical decisions. Progress in this field depends on collaboration, especially in developing shared datasets and repositories for training reliable AI systems.

AI has the potential to enhance diagnosis, treatment planning, and therapy optimization, but public awareness of its significance in cancer research and oncology is still limited. Educating the public about how AI may help clinicians diagnose cancer earlier, predict outcomes, and personalize therapy is critical for gaining trust and acceptance. Increased awareness can boost community support, facilitate patient participation in AI‐driven trials, and ultimately hasten the inclusion of these technologies into standard cancer practice.

While tremendous progress has been achieved in using AI and MM for cancer diagnosis and treatment, major challenges remain in terms of interpretability, data quality, model generalizability, and practical application. Future advancements will most likely be driven by explainable AI, multimodal data integration, hybrid mechanistic‐data‐driven models, and patient‐specific digital twin technologies. Addressing these difficulties through interdisciplinary collaboration and rigorous clinical validation will be critical for transforming computational discoveries into dependable and effective cancer care solutions.

## Author Contributions

**Mohsin Kamran:** conceptualization, writing – original draft, formal analysis, methodology. **Abdul Majeed:** data curation, visualization, investigation, writing – review and editing. **A S M Rafiul Haque:** conceptualization, validation, supervision, writing – review and editing. **Johari Yap Abdullah:** supervision, project administration, writing – review and editing.

## Funding

The authors have nothing to report.

## Ethics Statement

The authors have nothing to report.

## Consent

The authors have nothing to report.

## Conflicts of Interest

The authors declare no conflicts of interest.

## Transparency Statement

“A S M R. Haque” affirms that this manuscript is an honest, accurate, and transparent account of the study being reported; that no important aspects of the study have been omitted; and that any discrepancies from the study as planned (and, if relevant, registered) have been explained.

## Supporting information


Supporting File 1

Supporting File 2

Supporting File 3

Supporting File 4

Supporting File 5

Supporting File 6

## Data Availability

Data sharing is not applicable to this article because no new datasets were generated or analyzed during the current study. This study is based on a comprehensive review of previously published literature.
