# Supplementary material for: Advancing Tumor Treatment Through Artificial Intelligence and Mathematical Modeling: A Comprehensive Review
Source: Health Sci Rep. 2026 Jul 27;9(8):e72884. doi: 10.1002/hsr2.72884 (PMC13403053; doi:10.1002/hsr2.72884)
Supplement: Supplementary file 1 — Supporting File 1 [file HSR2-9-e72884-s006.docx]

**Supplementary Figure 1**


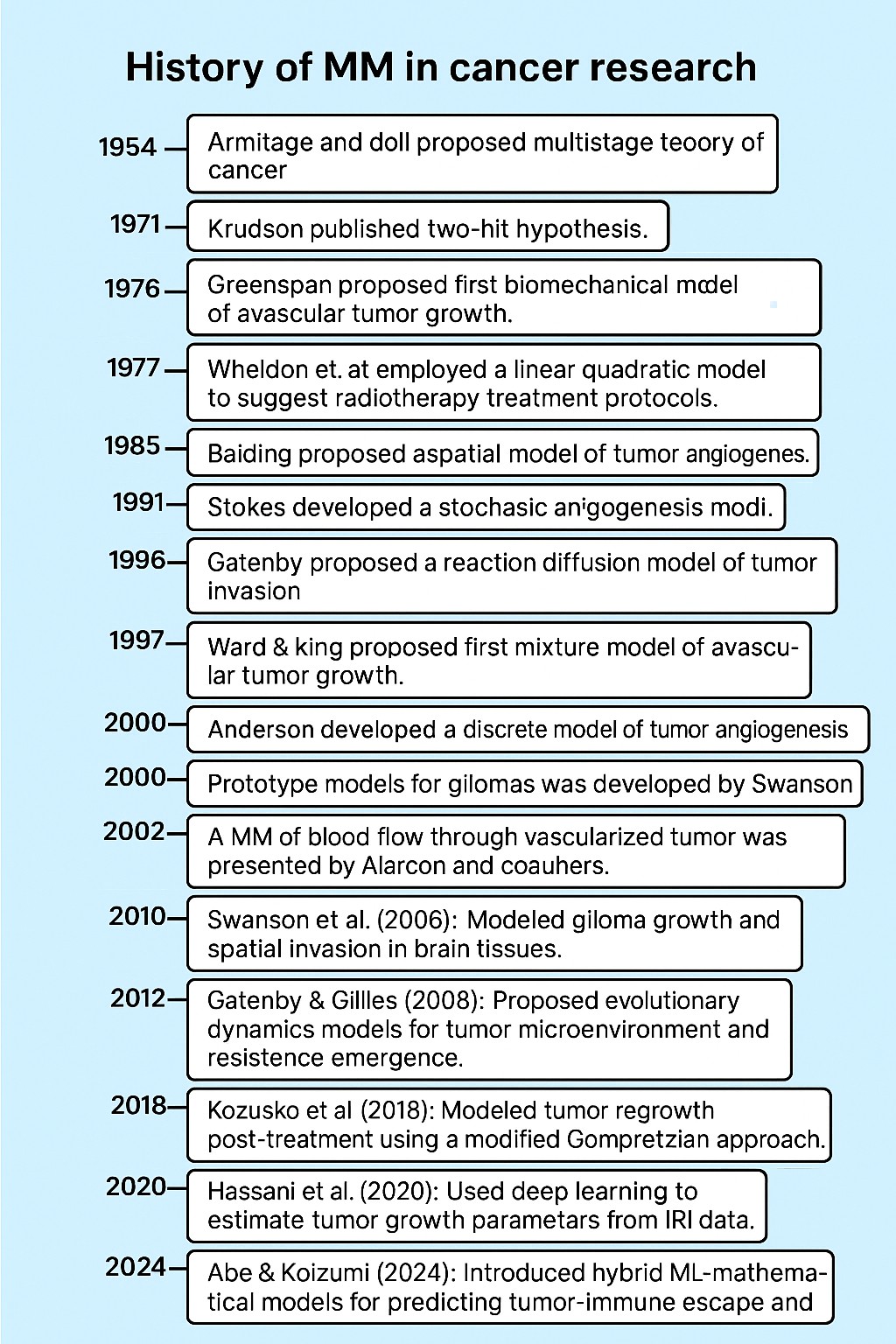


**Supplementary Figure 1:** History of mathematical modeling in cancer research
