# Supplementary material for: Advancing Tumor Treatment Through Artificial Intelligence and Mathematical Modeling: A Comprehensive Review
Source: Health Sci Rep. 2026 Jul 27;9(8):e72884. doi: 10.1002/hsr2.72884 (PMC13403053; doi:10.1002/hsr2.72884)
Supplement: Supplementary file 2 — Supporting File 2 [file HSR2-9-e72884-s001.docx]

**Supplementary Table 1**

**Table 1**: Differences between cancerous and non-cancerous tumor

| **Trait** |  | |
| --- | --- | --- |
|  | **Benign** | **Malignant** |
| Nuclear Size | Small | Large |
| Ratio of nuclear size to cytoplasmic volume | Low | High |
| Nuclear shape | Regular | Pleomorphic (irregular shape) |
| Mitotic index | Low | High |
| Tissue organization | Normal | Disorganized |
| Differentiation | Well differentiation | Poorly differentiated |
| Tumor boundary | Well defined | Poorly defined |
