# Supplementary material for: Advancing Tumor Treatment Through Artificial Intelligence and Mathematical Modeling: A Comprehensive Review
Source: Health Sci Rep. 2026 Jul 27;9(8):e72884. doi: 10.1002/hsr2.72884 (PMC13403053; doi:10.1002/hsr2.72884)
Supplement: Supplementary file 3 — Supporting File 3 [file HSR2-9-e72884-s005.docx]

**Supplementary Table 2**

**Table 2**: Comparison of AI Models in Cancer Prediction

| **Model Type** | **Techniques Used** | **Strengths** | **Limitations** | **Applications** |
| --- | --- | --- | --- | --- |
| Artificial Neural Networks (ANN) | Deep learning, multi- layer perceptron | High accuracy, handles  nonlinear data | Black-box nature, overfitting | Tumor classification, imaging |
| Support Vector Machines (SVM) | Kernel-based classifi- cation | Effective in small datasets,  robust | Sensitive to parameter tuning | Cancer diagnosis, biomarker analysis |
| Random Forest (RF) | Ensemble learning | Handles high-dimensional data, reduces overfitting | Less interpretable | Genomic data analysis |
| Hybrid Models | ANN + Optimization  / ML combinations | Improved accuracy, robustness | Increased complexity | Multi-cancer prediction |
