# Supplementary material for: Advancing Tumor Treatment Through Artificial Intelligence and Mathematical Modeling: A Comprehensive Review
Source: Health Sci Rep. 2026 Jul 27;9(8):e72884. doi: 10.1002/hsr2.72884 (PMC13403053; doi:10.1002/hsr2.72884)
Supplement: Supplementary file 4 — Supporting File 4 [file HSR2-9-e72884-s003.docx]

**Supplementary Table 3**

**Table 3**: Comparative analysis of ML and DL approaches for multi-cancer detection based on reviewed literature

| **Ref.** | **Cancer Type** | **Method** | **Acc.** | **Sens.** | **Spec.** | **AUC** | **Prec.** | **F1** |
| --- | --- | --- | --- | --- | --- | --- | --- | --- |
| [66] | Multi-cancer | ML (SVM, RF, ANN) | High | – | – | – | – | – |
| [65] | Multi-cancer | ML Classification | High | – | – | – | – | – |
| [68] | Multi-cancer | Deep Learning (CNN) | High | High | High | – | – | – |
| [70] | Multi-cancer | Deep Neural Networks | High | – | – | – | – | – |
| [8–16] | Lung / Brain | Hybrid Modeling + ML | Improved | – | – | – | – | – |
| [131] | Multi-cancer | ANN, SVM | 91–95% | – | – | – | – | – |
| [132] | Breast | SVM, LR, KNN | 85–97% | – | – | – | – | – |
| [133] | Breast | ML models | – | 0*.*87 | 0*.*90 | 0*.*92 | – | – |
| [134] | Breast | RF, SVM, DT | 90–98% | – | – | – | – | – |
| [151] | Skin | Ensemble CNN | 93% | 0*.*90 | 0*.*94 | 0*.*95 | 0*.*91 | 0*.*90 |
| [181] | Multi-cancer | Hybrid DL | 92% | 0*.*88 | 0*.*93 | 0*.*94 | 0*.*90 | 0*.*89 |
| [254] | Brain (MRI) | ML on Imaging Data | Improved | – | – | – | – | – |
| [255] | Surgical Data | ML-based Prediction | Moderate | – | – | – | – | – |
| [257] | Multi-domain | ML in Imaging | High | – | – | – | – | – |
| [259–262] | Multi-cancer | Advanced DL (Feature Learning) | High | High | High | – | – | – |
| Recent DL Studies (2024–2025) | Multi-cancer | CNN / Transformers | 90–99% | High | High | 0*.*90–0*.*97 | High | High |
