# Supplementary material for: Advancing Tumor Treatment Through Artificial Intelligence and Mathematical Modeling: A Comprehensive Review
Source: Health Sci Rep. 2026 Jul 27;9(8):e72884. doi: 10.1002/hsr2.72884 (PMC13403053; doi:10.1002/hsr2.72884)
Supplement: Supplementary file 5 — Supporting File 5 [file HSR2-9-e72884-s002.docx]

**Supplementary Table 4**

**Table 4:** Summary of datasets for cancer research

| **Database/service/project** | **Lab** | **No & Type of Cancer(s)** | **Accessed in** | **Description** |
| --- | --- | --- | --- | --- |
| TCGA | [https://www.cancer.gov/aboutnci/organization/ccg/](http://www.cancer.gov/aboutnci/organization/ccg/) research/structural-genomics/tcga | 33, Multiple | September 24*,* 2021 | 11125 |
| Rotterdam tumor bank | https://stat.ethz.ch/R-manual/R-devel/library/survival/ html/rotterdam.html | Breast Cancer | September 24*,* 2021 | Out of 2982 individuals with primary breast cancer, 1546 were posi- tive. |
| SUPPORT | [188] | 9105, Multiple | September 24*,* 2021 | Over a 6-month period, 47% of 9105 adults died. |
| METABRIC | [https://www.cbioportal.org/study/summary?id=brcametabric](http://www.cbioportal.org/study/summary?id=brcametabric) | Breast Cancer | September 24*,* 2021 | There were 2509 primary breast tumor patients and 548 normal con- trol participants. |
| MITOS-ATYPIA-14 | https://mitos-atypia-14.grand-challenge.org/Home/ | Breast cancer | September 24*,* 2021 | 1539 *×* 1376 pixels of resolution at 20x and 40x magnification levels. |
| TUPAC 2016 | [189] | Breast cancer | September 24*,* 2021 | 321 test and 500 training breast cancer histology wholebrain slides were used. |
| INbreast | [190] | 115, Breast cancer | September 24*,* 2021 | 410 images |
| LIDC-IDRI | https://wiki.cancerimagingarchive.net/display/Public/LIDCIDRI1966254194132fe653e4a7db00715f6f 775c012 | Lung cancer | September 24*,* 2021 | CT scans of 1018 patients were divided into three categories: nodules (= 3 mm), nodules (¡ 3 mm) and nonnodules (= 3 mm). |
| LUNA16 | https://luna16.grandchallenge.org/Data/ | Lung cancer | September 24*,* 2021 | 888 CT scans enable segmentation investigations. |
| BreakHis | https://web.inf.ufpr.br/vri/databases/breastcancer histopathologicaldatabasebreakhis/ | Breast cancer | September 24*,* 2021 | 9109 microscopic pictures at four distinct magnification levels (40x,  100x, 200x and 400x) were obtained from 82 patients. |
| CAMELYON | https://camelyon17.grand-challenge.org | Breast cancer | September 24*,* 2021 | Facilitates patient-level analysis; 1399 unique whole-slide images; no metastases, macrometastases, micrometastases and isolated tumor cell |
| MITOS12 | <http://ludo17.free.fr/mitos2012/dataset.html> | Breast cancer | September 24*,* 2021 | 50 biopsy slides; 40x magnification level; more than 300 mitoses |
| Leukemia microarray gene | [https://www.bioconductor.org/packages/devel/data/experiment/manuals/leukemiasEset/man/](http://www.bioconductor.org/packages/devel/data/experiment/manuals/leukemiasEset/man/) leukemias- Eset.pdf | Bone marrow cancer | September 24*,* 2021 | 60 bone marrow samples; acute lymphoblastic leukemia, acute myeloid leukemia, chronic lymphocytic leukemia, chronic myeloid leukemia and healthy bone marrow |
| PatchCamelyon | [https://www.tensorflow.org/datasets/catalog/patchcamelyon](http://www.tensorflow.org/datasets/catalog/patchcamelyon) | Breast cancer | September 24*,* 2021 | The dataset contains 327*,* 680 color images with a size of 96 *×* 96 pixels, making it larger than CIFAR10 but smaller than ImageNet. |

| **Database/service/project** | **Lab** | **No & Type of Cancer(s)** | **Accessed in** | **Description** |
| --- | --- | --- | --- | --- |
| 2018 ICIAR | https://iciar2018-challenge.grand-challenge.org/Dataset/ | Breast cancer | September 24*,* 2021 | Normal, benign, in situ cancer and aggressive carcinoma are repre- sented by 400 microscope images, 100 in each class. |
| UCSB-BB | https://bioimage.ucsb.edu/research/biosegmentation | Supports breast cancer research in human | September 24*,* 2021 | Image collection includes subcellular, cellular and tissue photos of hu- mans, monkeys and cats. |
| BRATS | [https://www.med.upenn.edu/cbica/brats2020/](http://www.med.upenn.edu/cbica/brats2020/) | Brain tumor | September 24*,* 2021 | MRI scans of 65 participants in both clinical and synthetic datasets were used for brain tumor segmentation. |
| Kvasir | https://dl.acm.org/do/10.1145/3193289/abs/ | Gastrointestinal tract cancer | September 24*,* 2021 | 4000 annotated photos in 8 classifications. |
| GDC | https://gdc.cancer.gov | Multiple | September 24*,* 2021 | Data on genomics, clinical trials and biospecimens are provided. |
| TARGET | https://ocg.cancer.gov/programs/target | Multiple | September 24*,* 2021 | This study supports childhood cancers and gives extensive genomic data to assess molecular changes. |
| 2015 Bioimaging Breast Histology Classification Challenge | https://rdm.inesctec.pt/dataset/nis-2017-003 | Breast cancer | September 24*,* 2021 | The image has a size of 2040 *×* 1536 pixels and is divided into four categories: normal, benign, in situ and invasive. |
| 1000 Genomes Project | [https://www.internationalgenome.org/1000genomessummary](http://www.internationalgenome.org/1000genomessummary) | Multiple | September 24*,* 2021 | Offers detailed information on human genetic variation. |
| Gene Expression Omnibus repository | [https://www.ncbi.nlm.nih.gov/geo/](http://www.ncbi.nlm.nih.gov/geo/) | Multiple | September 24*,* 2021 | Offers extensive genetic data sets, including microarrays and next- generation sequencing. |
| BioGPS | [http://biogps.org/goto=welcome](http://biogps.org/goto%3Dwelcome) | Multiple | September 24*,* 2021 | Supports eight species, including humans and several forms of cancer. |
| TCIA | [https://www.cancerimagingarchive.net](http://www.cancerimagingarchive.net/) | Multiple | September 24*,* 2021 | Supports several modalities and data types, including patient outcomes, treatment details and genomes. |
