# Supplementary material for: Advancing Tumor Treatment Through Artificial Intelligence and Mathematical Modeling: A Comprehensive Review
Source: Health Sci Rep. 2026 Jul 27;9(8):e72884. doi: 10.1002/hsr2.72884 (PMC13403053; doi:10.1002/hsr2.72884)
Supplement: Supplementary file 6 — Supporting File 6 [file HSR2-9-e72884-s004.docx]

**Supplementary Table 5**

**Table 5:** Growth models describing tumor dynamics

| **Models/Assumptions** | **Equation** |
| --- | --- |
| Linear Growth | $T^{'}\left( t \right)=\kappa_{g}, T^{'}\left( t \right)=\kappa_{g}-\delta T$ |
| Exponential Growth | $T^{'}\left( t \right)=\kappa_{g}T, T^{'}\left( t \right)=\kappa_{g}T-\delta T$ |
| Logistic Growth | $T^{'}\left( t \right)=\kappa_{g}T\left( 1-\frac{T}{T_{max}} \right)$ |
| Gompertz Growth | $T^{'}\left( t \right)=\kappa_{g}T\ln\left( \frac{T_{max}}{T} \right), \frac{d(\ln T)}{dt}=a-b ln T$ |
| Combined exponential and linear growth | $T^{'}\left( t \right)=\lambda_{0}T\left( \left[ 1+\left( T\frac{\lambda_{0}}{\lambda_{1}} \right)^{20} \right]^{\frac{1}{20}} \right)^{-1}$ |
| Tumor heterogeneity (Proliferative + Quiescent) | $P^{'}=g\left( P \right)-n_{1}P+n_{2}Q$  $Q^{'}=n_{1}P-n_{2}Q$ |
| Sensitive + Resistant tumor burden | $S^{'}=g\left( S \right)-n_{1}S+n_{2}R$  $R^{'}=g\left( R \right)+n_{1}S-n_{2}R$ |
| Angiogenesis model | $T^{'}=\kappa_{g}T\left( 1-\frac{T}{E} \right), E^{'}= \kappa_{2}T^{0.5}$ |
| Immune Interaction (Model 1) | $T^{'}=g\left( T \right)-g\left( I \right)T\left( \frac{h}{T+h} \right)$  $g\left( I \right)=\left( \delta_{1}I_{1}+\delta_{2}I_{2} \right)\left( \frac{I_{3}}{f+I_{3}} \right)$ |
| Immune Interaction (Model 2) | $T^{'}=g\left( T \right)-\delta_{1}IT-\delta_{2}NT$  $I^{'}=g\left( I \right)-\delta IT$ |
| Two-phase algebraic model | $T=\left( e^{-\kappa_{d}t}+e^{\kappa_{g}t}-1 \right).BASE$ |
| Algebraic model | $T=\left( {\varphi e}^{-\kappa_{d}t}+(e^{\kappa_{g}t}-\varphi) \right).BASE$ |
| Wang model | $T=BASE.e^{-At}+Bt+Ct^{2}$ |
| Reaction-diffusion model | $\frac{\partial C}{\partial t}=\mathrm{Dif}.\nabla^{2}C+f\left( C \right)-\left( 1-\mathrm{Surv} \right)f(C)$ |
| Proliferation-invasion model | $\frac{\partial C}{\partial t}=\mathrm{Dif}.\nabla^{2}C+f\left( C \right)-G(x,t)$ |
